# An Endophytic *Bacillus amyloliquefaciens* ZN-S18 Suppresses *Fusarium oxysporum* f. sp. *lycopersici* Through Synergistic Activity of Surfactin and Iturin A

**DOI:** 10.3390/jof12080576

**Published:** 2026-08-04

**Authors:** Dan-Na Ma, Yihan Niu, Mengli Chen, Jun-Nan Yao, Huiming Wu, Taiying Li

**Affiliations:** 1 College of Advanced Agriculture Science, Zhejiang A&F University, Hangzhou 311300, China; 17816046074@163.com (D.-N.M.); niuyihan03@126.com (Y.N.);; 2 Zhejiang Key Laboratory of Biology and Ecological Regulation of Crop Pathogens and Insects, Hangzhou 311300, China

**Keywords:** biological control, cell wall integrity, cyclic lipopeptides, endophyte, synergistic effect, tomato fusarium wilt

## Abstract

Fusarium wilt, caused by the soil-borne fungus *Fusarium oxysporum* f. sp. *lycopersici* (Fol), is a devastating disease that severely constrains tomato production worldwide. In this study, an endophytic bacterium, designated ZN-S18, was isolated from healthy tomato stems and identified as *Bacillus amyloliquefaciens* based on phylogenetic analyses of the 16S rRNA and *rpoB* genes. Dual culture assays demonstrated that ZN-S18 exhibited strong antagonistic activity against Fol and significantly inhibited fungal mycelial growth. The cell-free culture filtrate of ZN-S18 markedly suppressed Fol spore germination and mycelial growth, and the inhibitory effect on spore germination was irreversible. LC-MS analyses confirmed that ZN-S18 produces the cyclic lipopeptides surfactin and iturin A. In planta assays further demonstrated that pretreatment with ZN-S18, its culture filtrate, or the combination of surfactin and iturin A significantly reduced Fusarium wilt in tomato seedlings. Moreover, the culture filtrate disrupted fungal cell wall integrity, as indicated by the increased sensitivity of Fol to Congo red stress. Notably, the combined application of surfactin and iturin A exhibited significantly stronger antifungal activity than either compound alone under Congo red-containing conditions, suggesting a synergistic effect on fungal cell wall integrity. Collectively, these findings indicate that the endophytic strain *B. amyloliquefaciens* ZN-S18 is a promising biocontrol agent against tomato Fusarium wilt, primarily through the synergistic action of surfactin and iturin A. This study provides an environmentally friendly alternative to chemical fungicides and establishes a foundation for future investigations into the molecular mechanisms underlying lipopeptide-mediated antagonism.

## 1. Introduction

Tomato (*Solanum lycopersicum*) is one of the most widely cultivated horticultural crops worldwide and represents a major source of nutritional and economic value. As a member of the Solanaceae family, tomato is closely related to other economically important crops, including tobacco, potato, and pepper. Tomato fruits are rich in bioactive compounds such as β-carotene, vitamin C, lycopene, flavonoids, and hydroxycinnamic acid derivatives [1], all of which contribute substantially to human health and dietary quality. Owing to its high nutritional value and broad consumer demand, tomato cultivation has expanded globally, with China accounting for approximately 37% of global production.

Despite its agricultural importance, tomato production is severely threatened by a wide range of biotic stresses, including insect pests, nematodes, oomycetes, and fungal pathogens [2,3,4,5,6,7,8]. Among these diseases, Fusarium wilt caused by the soil-borne fungus *Fusarium oxysporum* f. sp. *lycopersici* (Fol) is one of the most destructive vascular diseases affecting tomato production worldwide [9,10,11]. Fol invades tomato roots and colonizes xylem vessels, leading to vascular discoloration, wilting, and eventually plant death. The pathogen can survive in soil for extended periods through the production of dormant chlamydospores and conidia, making disease management particularly challenging. Moreover, the progression of the pathogen from the root cortex into the xylem vessels represents a critical stage in disease development and complicates effective management of Fusarium wilt [12,13].

Chemical fungicides have long been used for management of Fol and other soil-borne pathogens [14,15]. Although chemical control strategies can provide short-term disease suppression, their excessive and prolonged use has resulted in environmental pollution, soil ecosystem disruption, and the emergence of fungicide-resistant pathogen populations. Consequently, there is increasing demand for environmentally sustainable and biologically based alternatives for disease management.

Biological control using antagonistic microorganisms has emerged as a promising strategy for the sustainable management of plant disease [16,17,18,19]. Numerous bacterial genera, including *Streptomyces*, *Pseudomonas*, and *Bacillus*, have been reported to suppress phytopathogens through multiple mechanisms, such as the production of antimicrobial compounds, competition for nutrients and ecological niches, and induction of host resistance [20]. Among them, *Bacillus* species have attracted considerable attention owing to their strong antagonistic activity [21,22,23], environmental adaptability, and ability to colonize plant tissues [24].

The antagonistic activity of *Bacillus* spp. is frequently associated with the production of cyclic lipopeptides and volatile organic compounds (VOCs) [25]. Cyclic lipopeptides such as surfactin, fengycin, and iturin are well-known antimicrobial metabolites that can disrupt fungal cell membranes and inhibit pathogen growth [26]. In addition, VOCs produced by antagonistic bacteria can inhibit fungal development through long-distance interactions [27]. In recent years, endophytic microorganisms residing within plant tissues have received increasing attention because of their ability to enhance plant resistance against pathogens while maintaining close associations with their hosts [28,29].

In this study, we isolated an endophytic bacterium from healthy tomato stems and evaluated its antagonistic activity against Fol. The isolate exhibited strong inhibitory effects on mycelial growth and spore germination and significantly reduced Fusarium wilt in tomato seedlings. Furthermore, molecular and chemical analyses demonstrated that the isolate produces surfactin and iturin A, which synergistically impair fungal cell wall integrity. These findings suggest that this endophytic bacterium has considerable potential as a biocontrol agent for the sustainable management of tomato Fusarium wilt.

## 2. Materials and Methods

### 2.1. Isolation of Endophytic Bacteria from Healthy Tomato Plants

Healthy tomato plants showing no symptoms of Fusarium wilt were collected for endophyte isolation. Stem segments were cut into slices and surface sterilized in 70% ethanol for 1 min, followed by treatment with 0.5% sodium hypochlorite for 20 min. The tissues were subsequently rinsed thoroughly with sterile water (SW). The sterilized stem segments were placed on Luria–Bertani agar (LBA) and incubated at 28 °C for 2 d. Emerging bacterial colonies were purified by repeated streaking on fresh LBA. Purified isolates were stored in 50% glycerol at −80 °C.

### 2.2. Dual Culture Assay

The antagonistic activity of bacterial isolates against Fol was evaluated using dual culture assays. Fol was cultured on complete medium (CM) at 25 °C for 3 d, whereas bacterial isolates were cultured on LBA at 28 °C for 1 d. A mycelial plug of Fol was placed at the center of a CM, and bacterial isolates were inoculated on both sides of the fungal plug. Plates were incubated at 28 °C for 3 d, and the isolate showing the strongest inhibitory activity was selected for further analyses.

To determine the optimal medium for antagonistic activity, dual culture assays were additionally conducted on CM, potato dextrose agar (PDA), and minimal medium (MM). *Pseudomonas syringae* and *Escherichia coli* were used as controls.

### 2.3. Identification of Antagonistic Endophyte

Phylogenetic analyses were performed using the 16S rRNA and *rpo B* gene sequences [30]. Sequence alignments were generated using MEGA X software, and phylogenetic trees were constructed using the maximum likelihood (ML) method with 1000 bootstrap replications [31]. Concatenated analyses of the 16S rRNA and *rpoB* sequences were also performed to improve taxonomic resolution. *E. coli* and *Burkholderia glumae* were used as outgroups.

### 2.4. Evaluation of Effects of Culture Filtrate on Spore Germination and Mycelial Growth of Fol

*Fusarium oxysporum* f. sp. *lycopersici* was cultured in CM broth at 25 °C with shaking at 180 rpm for 3 d. Fungal mycelia were harvested by filtration and transferred onto yeast malt agar (YMA) medium, followed by incubation under near-UV light at room temperature for 2 d to induce sporulation. Spores were collected by filtration through Miracloth and centrifugation at 13,000 rpm for 10 min. ZN-S18 was cultured in CM broth at 28 °C with shaking at 180 rpm for 3 d. The culture was centrifuged at 6000 rpm for 20 min, and the supernatant was filtered through a 0.45 µm syringe filter to obtain cell-free culture filtrate.

For spore germination assays, the culture filtrate was mixed with fresh CM broth at a ratio of 1:1 (*v*/*v*), and Fol spores were added to a final concentration of 1 × 10^6^ spores/mL. The suspensions were incubated at 28 °C with shaking at 180 rpm, and spore germination was evaluated at 4 h intervals. SW was used instead of culture filtrate in the mock.

For the mycelial growth assay, the culture filtrate was concentrated 10-fold by freeze-drying and resuspension in SW. An aliquot (100 µL) was evenly spread onto CM and allowed to dry under sterile conditions. A Fol mycelial plug was then inoculated onto the center of each plate and incubated for 4 d before colony diameter was measured. SW-treated CM served as the mock. Three biological replicates were used for all assays.

### 2.5. Evaluation of Fusarium Wilt Control Efficacy

Tomato seeds (cv. Diana) were surface-sterilized with 70% ethanol for 1 min, followed by 0.5% sodium hypochlorite for 20 min, and then rinsed twice with SW. Seeds were germinated at 25 °C, and 7-d-old seedlings were transplanted into vermiculite, and 4 mL of SW was added to each seedling.

Seedlings were divided into four groups: Mock, ZN-S18, Fol, and Fol + ZN-S18. All treatments were applied by root irrigation. After 2 d of acclimation, the Mock and Fol groups received 1 mL SW, whereas the ZN-S18 and Fol + ZN-S18 groups received 1 mL bacterial suspension (OD_600_ = 1.0). Two days later, the Mock and ZN-S18 groups were treated with SW, while the Fol and Fol + ZN-S18 groups were inoculated with 1 mL spore suspension (1 × 10^6^ spores/mL). Treatments were repeated weekly for 30 d.

Plants were maintained in a growth chamber at 25 °C under 100% relative humidity and a 16 h light/8 h dark photoperiod. Disease symptoms were recorded photographically. In addition, the disease suppression efficacy of the culture filtrate and the combination of 179 mg/L surfactin (CAS 24730-31-2, Aladdin, Shanghai, China) and 11 mg/L iturin A (CAS 52229-90-0, Psaitong Biotechnology, Beijing, China) was also evaluated. All experiments were conducted independently three times. For the bacterial treatment, each treatment included three replicates, whereas the culture filtrate and cyclic lipopeptide treatments each included two replicates. Disease severity was scored on a 0–4 scale as follows: 0, no disease symptoms; 1, slight yellowing or abnormal growth; 2, moderate chlorosis or wilting; 3, severe wilting or stunted growth; 4, the whole plant wilted or dead [32,33].

### 2.6. Verification of Antimicrobial Compounds

Based on the previously reported antimicrobial biosynthetic genes [34,35,36], PCR assays were performed to detect genes associated with cyclic lipopeptide biosynthesis in ZN-S18. PCRs were conducted in 20 µL volumes containing 10 µL 2 × Rapid Taq Master Mix (Vazyme, Jiangsu, China), 0.8 µL genomic DNA, 0.8 µL of each primer, and 8.4 µL SW.

PCR amplification was performed under the following conditions: initial denaturation at 94 °C for 3 min, followed by 35 cycles of 94 °C for 1 min, 60 °C for 30 s, and 72 °C for 1 min, with a final extension at 72 °C. PCR products were separated on 1% agarose gels containing 0.01% Ultra GelRed (Vazyme, Jiangsu, China) and visualized under UV illumination. The target genes and primers are listed in Table 1.

For chemical verification, 50-fold concentrated culture filtrate and standard compound were subjected to LC-MS analysis (Fuda Testing Group, Shanghai, China).

### 2.7. Evaluation of Cell Wall and Cell Membrane Integrity

Cell wall and membrane integrity assays were conducted using Congo red (CR) and sodium dodecyl sulfate (SDS), respectively. CM were supplemented with CR (0, 25, 50, 75, and 100 mg/L) or SDS (0, 0.005, 0.01, 0.015, and 0.02%). Culture filtrate (100 µL) was evenly spread onto the plates and allowed to dry under sterile conditions. SW-treated plates served as mocks. Fol mycelial plugs were inoculated at the center of each plate and incubated at 25 °C for 3 d. Mycelial growth was measured and compared with that of the corresponding mocks. The cell wall integrity assay included five biological replicates per treatment and was repeated once, whereas the cell membrane integrity assay included five biological replicates per treatment and was repeated three times.

The effects of surfactin, iturin A, and their combination on Fol cell wall integrity were also evaluated using CM with or without 100 mg/L CR. Five biological replicates were included for each treatment.

### 2.8. Effects of ZN-S18 on the Other Phytopathogens

The antagonistic activity of ZN-S18 against *B. cinerea* and *Ralstonia solanacearum* was evaluated using dual culture assays. For *B. cinerea*, a mycelial plug was placed at the center of a CM, and ZN-S18 was inoculated on both sides. Plates were incubated at 25 °C for 3 d. For *R. solanacearum*, bacterial suspensions (OD_600_ = 0.1) were prepared after overnight culture in LB broth at 28 °C with shaking at 180 rpm. Following washing and resuspension in SW, 20 µL of each suspension was inoculated onto CM, casamino acids-peptone-glucose (CPG), and LBA at defined distances, followed by incubation at 28 °C for 2 d.

Volatile organic compounds were evaluated using partitioned plates separated by a 1 cm gap to prevent direct contact between microorganisms. Plates were sealed and incubated at 25 °C for 4 d. Fungal plugs near the initial inoculation sites were subsequently collected using a sterile cork borer (diameter = 7 mm) and transferred into 1 mL of SW. Suspensions were vortexed for 1 min, and spore concentrations were determined using a hemocytometer. The experiment was conducted independently three times with five biological replicates per experiment.

### 2.9. Statistical Analysis

Statistical analyses were performed using R software (version 4.5.1). Differences among multiple groups were analyzed using one-way analysis of variance (ANOVA), followed by Tukey’s honestly significant difference (HSD) test. Comparisons between two groups were conducted using Welch’s t-test. Differences were considered statistically significant at *p* < 0.05. Spore germination percentages and inhibition rates were arcsine square-root transformed prior to statistical analysis.

## 3. Results

### 3.1. Screening and Identification of the Endophyte with the Strongest Antagonistic Activity

**Figure 1:**
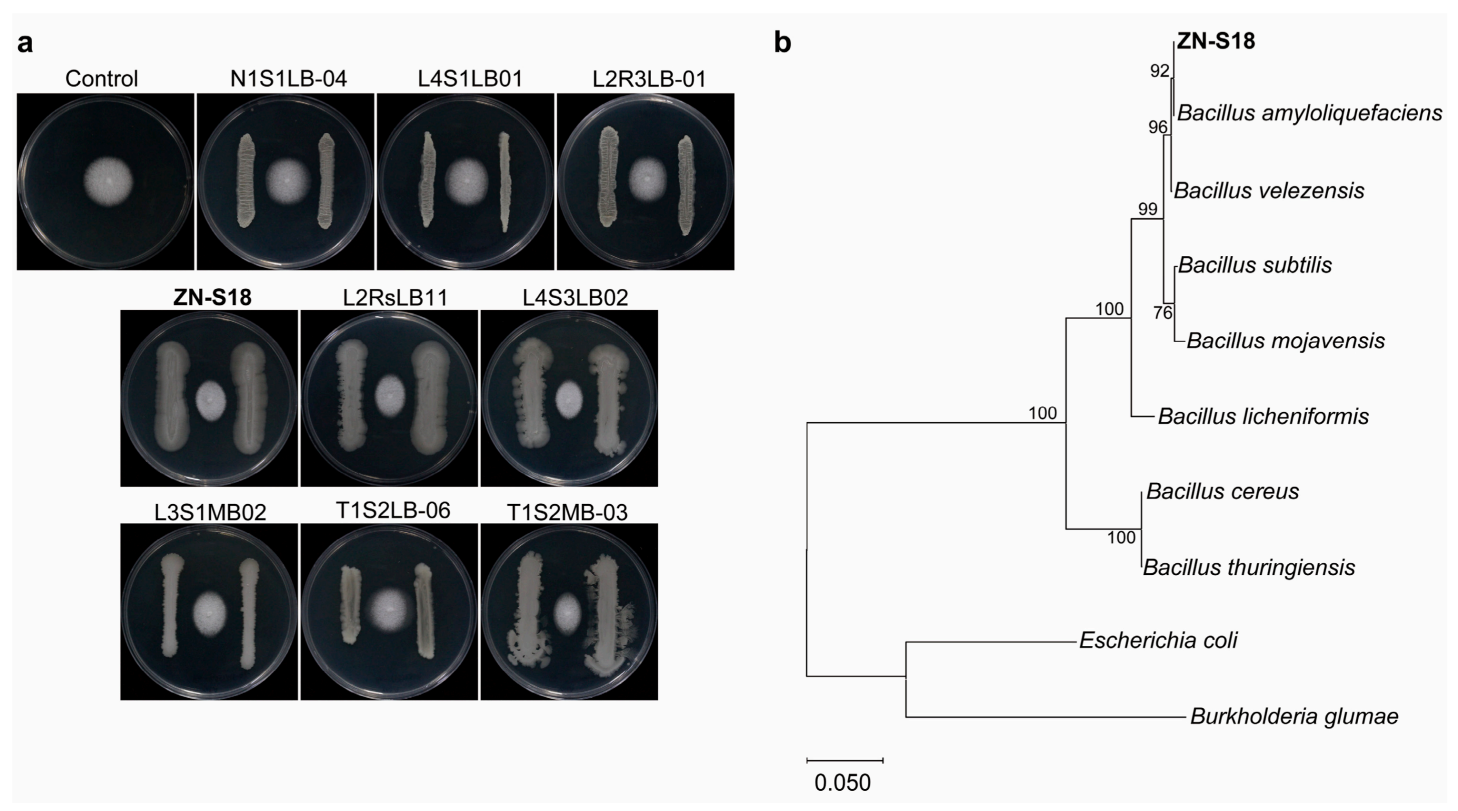
Antagonistic activity of bacterial isolates against *F. oxysporum* f. sp. *lycopersici* and phylogenetic analysis of ZN-S18. (**a**) The top nine bacterial isolates exhibiting antagonistic activity against *F. oxysporum* f. sp. *lycopersici* are shown. Each isolate was inoculated on both sides of the plate, whereas the fungal pathogen was placed at the center. Plates were incubated at 28 °C for 3 d. (**b**) A phylogenetic tree was constructed based on concatenated 16S rRNA and *rpoB* gene sequences. Sequence alignment was performed using ClustalW in MEGA X, and the tree was generated using the maximum likelihood method with 1000 bootstrap replications.

Several endophytic bacterial isolates were obtained from healthy tomato stems. Among them, strain ZN-S18 exhibited the strongest antagonistic activity against Fol in dual culture assays (Figure 1a). To identify this isolate, phylogenetic analyses based on the 16S rRNA and *rpoB* genes were performed. Both individual and concatenated phylogenetic analyses demonstrated that ZN-S18 clustered closely with *B. amyloliquefaciens* (Figure 1b and Figure S1). The 16S rRNA sequence was deposited in GenBank under accession number PV799877.

### 3.2. Effect of ZN-S18 on Fol Mycelial Growth Under Different Culture Conditions

Dual culture assays performed on CM, PDA, and MM demonstrated that ZN-S18 inhibited the mycelial growth of Fol on all tested media. The strongest inhibitory activity was observed on CM (Figure S2).

### 3.3. Effect of Secondary Metabolite of ZN-S18 on Fol

**Figure 2:**
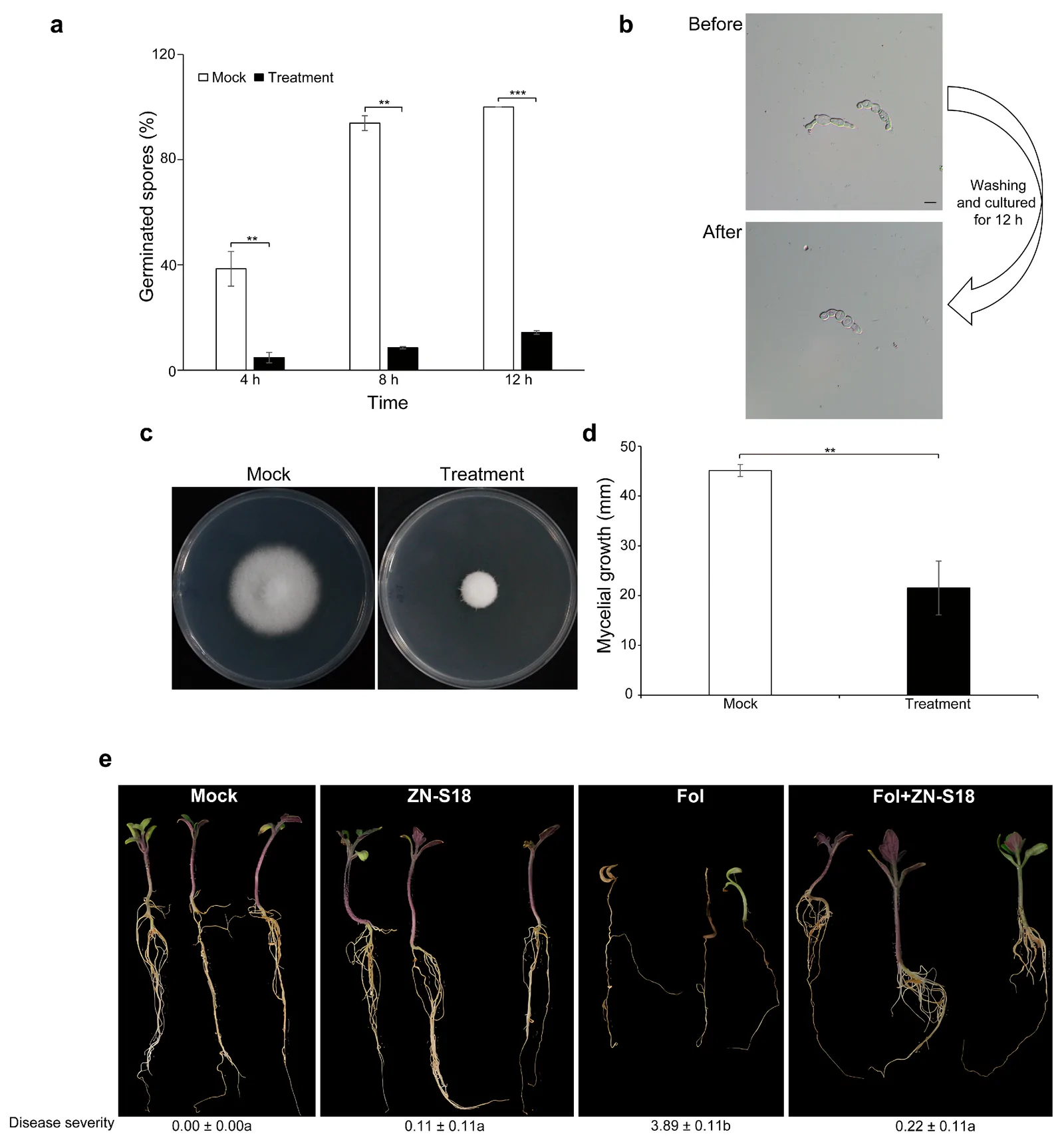
Effect of ZN-S18 culture filtrate on spore germination, mycelial growth of *F. oxysporum* f. sp. *lycopersici*, and its control efficacy against tomato Fusarium wilt. (**a**) Spore germination rates at 4, 8, and 12 h post-incubation at 25 °C. (**b**) Filtrate-treated spores were washed, resuspended in fresh CM broth, incubated for 12 h, and observed under a light microscope. Scale bars = 10 µm. (**c**,**d**) Mycelial growth on solid medium after 4 d at 25 °C. Mock plates did not contain culture filtrate. Error bars represent the standard deviation of the mean (*n* = 3). Asterisks indicate significant differences relative to the mock (** *p* < 0.01, *** *p* < 0.001). (**e**) Disease suppression assay in tomato seedlings. ZN-S18 (OD_600_ = 1.0) was applied by root irrigation 2 d prior to challenge with Fol (1 × 10^6^ spores/mL). Treatments were repeated weekly for 30 d. Plants were maintained at 25 °C under 100% relative humidity with a 16 h light/8 h dark photoperiod. The experiment was repeated three times with three biological replicates per treatment. Error bars represent the mean ± standard error (*n* = 3). Different letters indicate significant differences based on one-way ANOVA followed by Tukey’s HSD test (*p* < 0.05).

To determine whether ZN-S18 secretes antifungal metabolites, the effects of its cell-free culture filtrate on Fol spore germination and mycelial growth were evaluated. The culture filtrate significantly inhibited spore germination compared with the mock (Figure 2a). Furthermore, spores treated with the culture filtrate failed to recover germination after washing and transfer into fresh CM broth, indicating irreversible inhibition. Microscopic observations revealed abnormal swelling of treated spores (Figure 2b). Application of concentrated culture filtrate to solid medium also significantly reduced Fol mycelial growth compared with the mock (Figure 2c,d).

### 3.4. Effect of ZN-S18 on Fusarium Wilt Development in Tomato Seedlings

The biocontrol efficacy of ZN-S18 against tomato Fusarium wilt was evaluated in tomato seedlings. Treatment with ZN-S18 alone caused no visible disease symptoms, whereas Fol inoculation induced severe wilting symptoms in tomato seedlings. Pretreatment with ZN-S18 alleviated disease severity compared with Fol inoculation alone (Figure 2e). Similarly, pretreatment with either the culture filtrate or the combination of surfactin and iturin A reduced disease symptoms in tomato seedlings challenged with Fol (Figure S3).

### 3.5. Verification of Antifungal Metabolites Produced by ZN-S18

**Figure 3:**
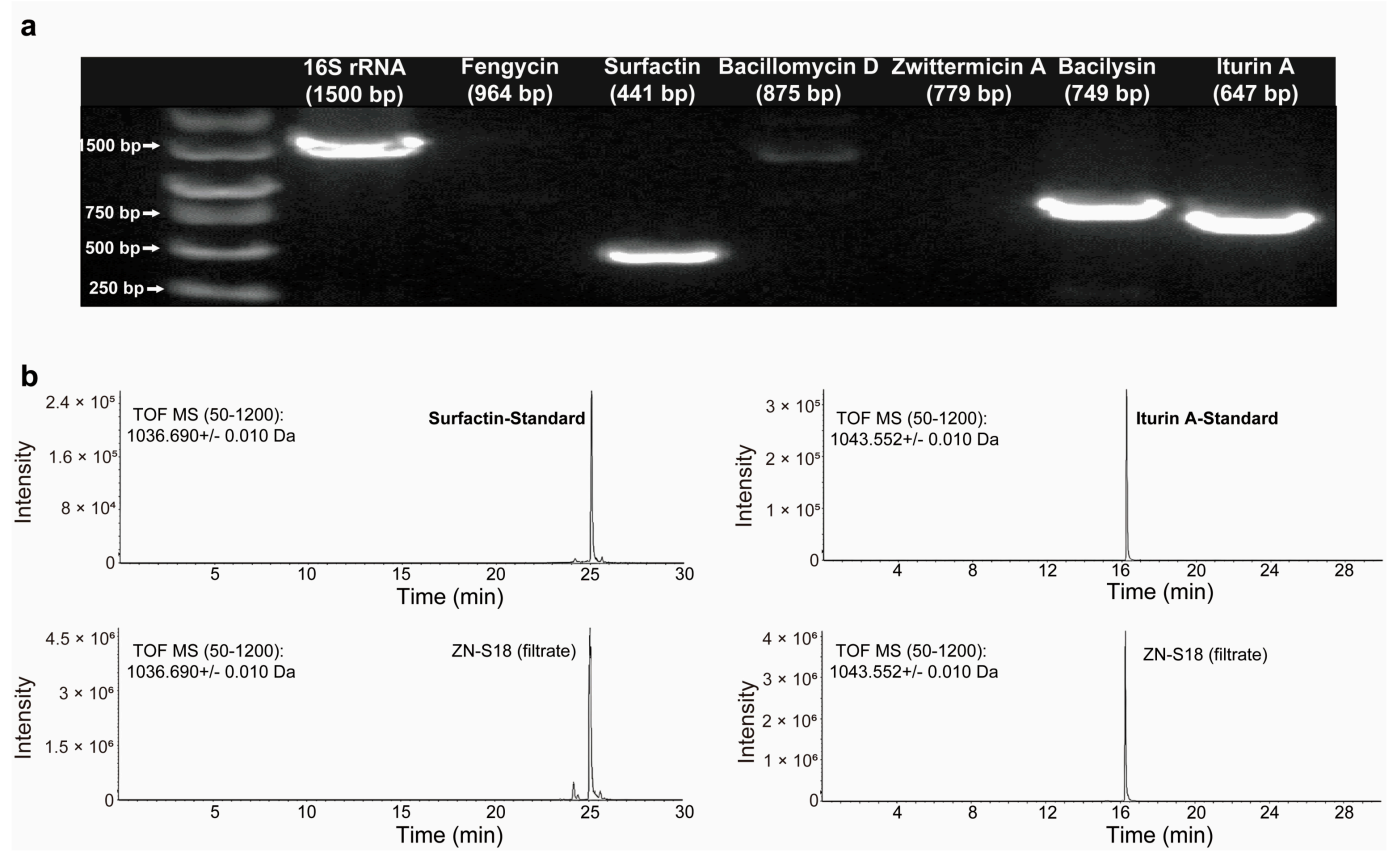
Identification of antifungal metabolites and related biosynthetic genes in ZN-S18. (**a**) PCR detection of genes associated with antimicrobial compound biosynthesis. (**b**) LC–MS analysis confirming the presence of antifungal compounds in the ZN-S18 culture filtrate. Extracted ion chromatograms of authentic standards and the culture filtrate are shown.

PCR analysis detected genes associated with the biosynthesis of surfactin, bacilysin, and iturin A in ZN-S18 (Figure 3a). To confirm metabolite production, the concentrated culture filtrate was analyzed by LC–MS. Extracted ion chromatograms revealed peaks at *m*/*z* 1036.690 and 1043.552 with retention times corresponding to surfactin and iturin A standards, respectively, confirming the production of these compounds by ZN-S18 (Figure 3b). In contrast, bacilysin was not detected in the culture filtrate.

### 3.6. Effect of ZN-S18 on Fol Cell Wall and Cell Membrane Integrity

**Figure 4:**
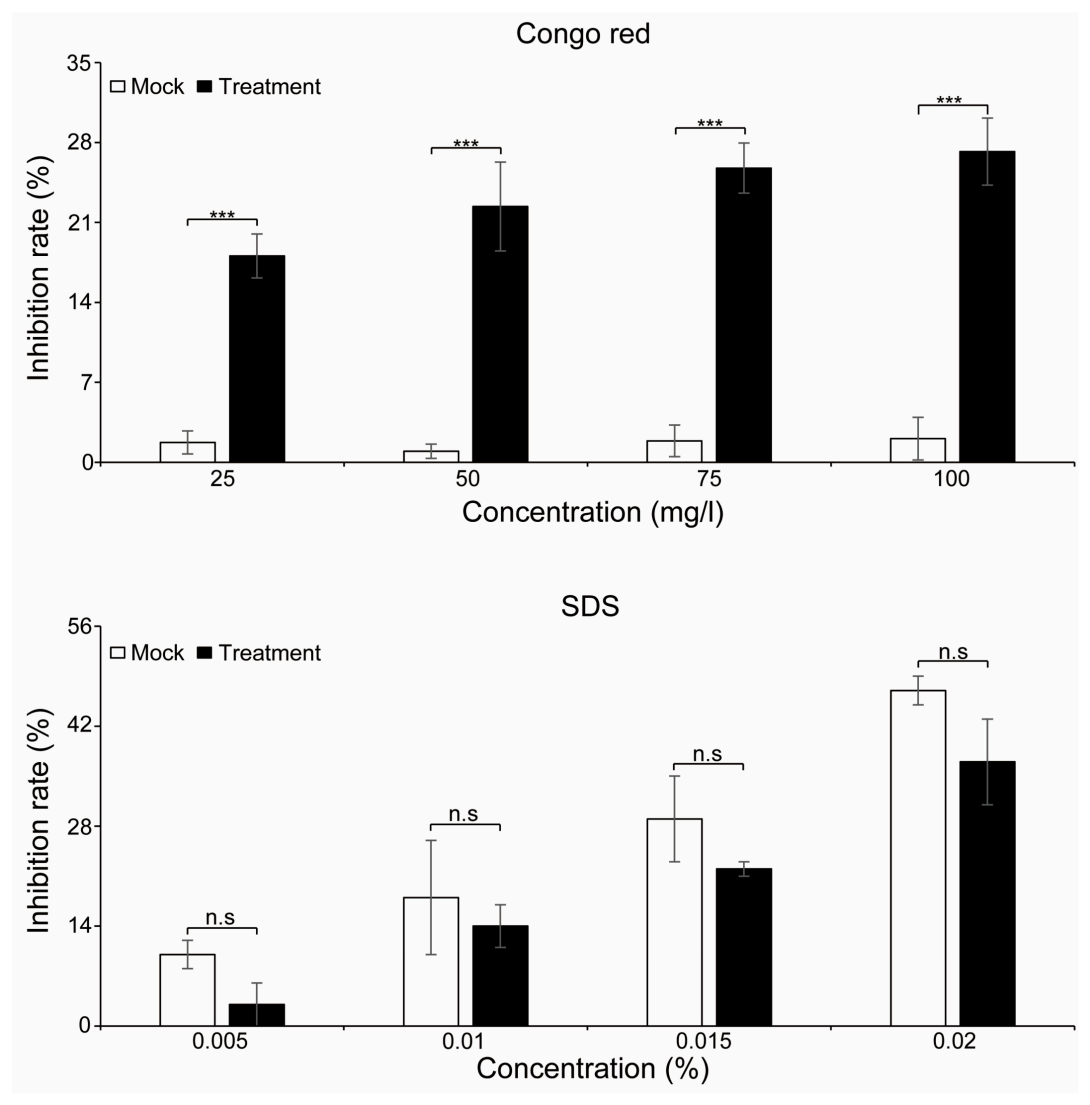
Effects of ZN-S18 culture filtrate on cell wall and membrane integrity of *F. oxysporum* f. sp. *lycopersici*. The fungus was cultured on CM supplemented with Congo red (0–100 mg/L) or SDS (0–0.02%), with or without ZN-S18 culture filtrate, and incubated at 25 °C for 3 d. For the Congo red assay, error bars represent the mean ± standard deviation (*n* = 5). For the SDS assay, error bars represent the mean ± standard error (*n* = 3). Asterisks indicate significant differences relative to the mock (*** *p* < 0.001); n.s. indicates no significant difference (*p* > 0.05).

Congo red and SDS assays were used to evaluate the effects of ZN-S18 metabolites on fungal cell wall and membrane integrity, respectively. Under CR stress conditions, the culture filtrate significantly inhibited Fol mycelial growth at all tested concentrations (Figure 4, upper panel). In contrast, no significant differences were observed under SDS stress conditions (Figure 4, lower panel).

**Figure 5:**
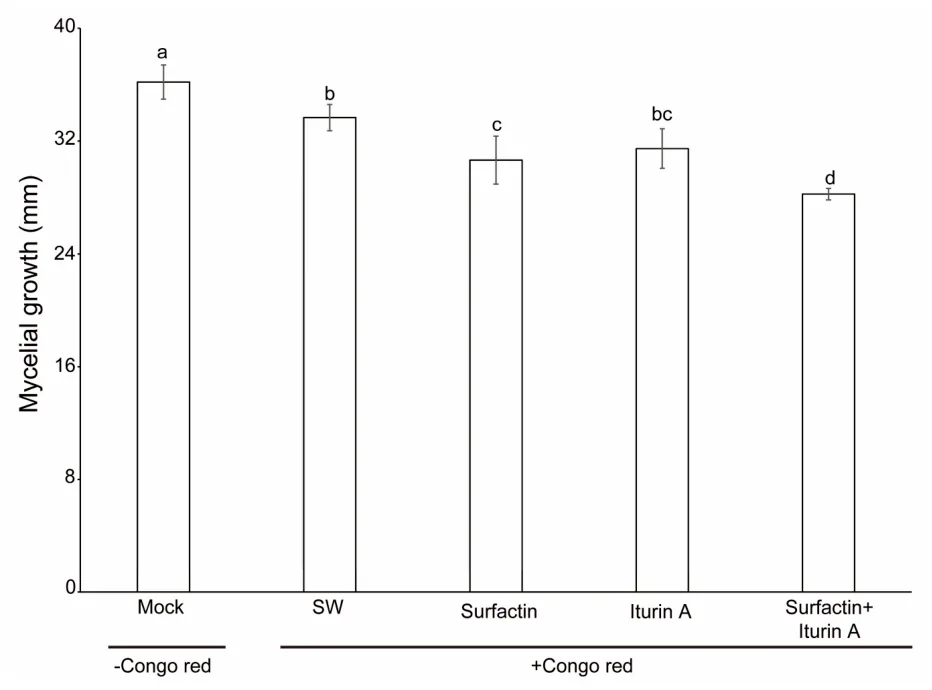
Synergistic effects of surfactin and iturin A on cell wall integrity of *F. oxysporum* f. sp. *lycopersici* under Congo red stress. The fungus was cultured on CM with or without 100 mg/L Congo red and treated with sterile water, surfactin alone, iturin A alone, or a combination of 179 mg/L surfactin and 11 mg/L iturin A for 3 d at 25 °C. Error bars represent the mean ± standard deviation (*n* = 5). Different letters indicate significant differences based on one-way ANOVA followed by Tukey’s HSD test (F = 31.1, *p* < 0.05).

The effects of surfactin, iturin A, and their combination were further investigated under CR stress. Surfactin significantly inhibited Fol mycelial growth, whereas iturin A alone exhibited relatively weak antifungal activity. Notably, the combined treatment showed significantly stronger inhibition than either compound alone (Figure 5).

### 3.7. Antagonistic Activity of ZN-S18 Against Other Phytopathogens

ZN-S18 also exhibited broad-spectrum antagonistic activity against other tomato pathogens. The strain effectively inhibited the growth of *R. solanacearum* on CM, CPG, and LBA. In dual culture assays, the mycelial growth of *B. cinerea* was severely restricted in the presence of ZN-S18 (Figure S4a,b). In addition, VOCs produced by ZN-S18 significantly inhibited both mycelial growth and spore production of Fol and *B. cinerea* (Figure S4c,d).

## 4. Discussion

Endophytic bacteria are widely distributed in plant tissues and are increasingly recognized for their capacity to promote plant growth and serve as effective biocontrol agents, thereby contributing substantially to sustainable agricultural production [37,38]. In the present study, we isolated an endophytic bacterium, strain ZN-S18, from the stems of healthy tomato plants and identified it as *B. amyloliquefaciens* through phylogenetic analyses. Members of this species are well documented for their potent antagonistic activity against diverse phytopathogens and their ability to efficiently colonize plant tissues [37,39]. Previous studies have further demonstrated that *B. amyloliquefaciens* strains isolated from plant tissues can effectively suppress fungal pathogens such as *B. cinerea* and *Fusarium* spp., while also reducing disease incidence in various crops [40,41,42,43]. In line with these findings, ZN-S18 exhibited strong inhibitory activity against Fol and significantly suppressed mycelial growth across multiple culture conditions (Figure S2).

For most fungal pathogens, spores represent the primary propagules for dispersal and infection initiation, as successful germination is a prerequisite for host penetration [44,45,46]. In this study, the cell-free culture filtrate of ZN-S18 markedly inhibited Fol spore germination, with the inhibitory effect remaining irreversible even after removal of the filtrate (Figure 2a,b). The observed swelling of treated spores further suggests structural or physiological damage. Such irreversible inhibition has been documented in other systems, where antimicrobial compounds disrupt spore integrity and metabolic processes, ultimately preventing germination [47,48]. Moreover, the culture filtrate of ZN-S18 also suppressed vegetative mycelial growth of Fol. Collectively, these results indicate that the antifungal metabolites produced by ZN-S18 target critical cellular processes essential for spore viability and mycelial development.

It is well established that impairment in mycelial growth, spore germination, and sporulation is closely associated with reduced pathogenicity in fungal pathogens [46,49,50]. Consistent with this principle, pretreatment of tomato seedlings with ZN-S18 alleviated disease symptoms caused by Fol (Figure 2e). Obviously, ZN-S18 exhibited no obvious plant growth-promoting effects under the tested conditions, suggesting that its primary function in this pathosystem is associated with direct antagonistic activity.

In addition to competing with phytopathogens for ecological niches, the production of secondary metabolites constitutes a key competitive strategy for endophytes [37,38,51]. *B. amyloliquefaciens* is well known for producing a variety of antagonistic metabolites, particularly cyclic lipopeptides. These lipopeptides are typically classified into three major families: fengycin, surfactin, and iturin [52,53]. All three families have been widely reported to display strong inhibitory activity against a broad spectrum of phytopathogens. The fengycin family comprises multiple isomers that vary in fatty acid chain length, branching patterns, and amino acid composition [54]. The surfactin family, one of the most extensively studied, consists of a heptapeptide linked to a β-hydroxy fatty acid chain ranging from C12 to C16, exhibiting potent antimicrobial efficacy [55,56]. The iturin family includes homologs such as iturin A–E, mycosubtilin, and bacillomycin D, F, L, and LC, each comprising a heptapeptide linked to a β-amino fatty acid chain with lengths ranging from C14 to C17 [47,57].

In this study, molecular and chemical analyses confirmed that ZN-S18 produces surfactin and iturin A (Figure 3b). Previous research has demonstrated that surfactin can effectively inhibit the growth and pathogenicity of ginseng root rot pathogens, suppress tomato bacterial wilt, and control wheat Fusarium head blight by inducing programmed cell death in fungi [36,58,59]. Similarly, iturin A has been shown to inhibit spore germination of the rice blast pathogen, thereby reducing pathogenicity, and to suppress the growth of *Aspergillus niger* along with its mycotoxin production [60,61].

Mechanistically, surfactin and iturin A are known to interact with lipid bilayers, increasing membrane permeability, causing ion leakage, and ultimately leading to cell death [62,63,64]. These compounds can also trigger downstream effects, including oxidative stress and disruption of signaling pathways [65,66]. In our study, the culture filtrate significantly compromised Fol cell wall integrity, as evidenced by increased sensitivity to CR stress (Figure 4). This suggests that, in addition to membrane disruption, the metabolites may interfere with cell wall biosynthesis or structural maintenance processes that are critical for fungal growth and morphogenesis.

It is noteworthy that the combined application of surfactin and iturin A resulted in significantly stronger inhibition of mycelial growth compared with either compound alone (Figure 5). Synergistic interactions among lipopeptides have been reported previously, wherein different compounds target complementary cellular structures or pathways, thereby enhancing overall antifungal efficacy [53,67,68]. This synergistic effect likely underpins the robust biocontrol performance of ZN-S18 and underscores the importance of metabolite diversity in effective pathogen suppression. Importantly, the combination of surfactin and iturin A conferred protective effects similar to those of the culture filtrate (Figure S3), indicating that these secreted compounds are sufficient to mediate disease suppression.

Interestingly, ZN-S18 also produced VOCs that significantly suppressed both mycelial growth and spore production of the Fol and *B. cinerea* (Figure S4). VOC-mediated inhibition has been increasingly recognized as a key mechanism in microbial antagonism, particularly for long-distance interactions [27,69,70]. The ability of ZN-S18 to inhibit multiple pathogens, including the bacterial pathogen *R. solanacearum*, further highlights its broad-spectrum antimicrobial potential. Such multi-target activity is advantageous for biocontrol applications, as it may reduce the likelihood of resistance development in target pathogens [64]. These findings suggest a promising avenue for future research, namely, integrating the confirmed inhibitory effects of surfactin and iturin A with synergistic contributions of VOCs.

Taken together, this study identifies ZN-S18 as a potent antagonistic endophyte belonging to *B. amyloliquefaciens*, capable of suppressing Fol through multiple mechanisms, including inhibition of mycelial growth, spore germination, and overall pathogenicity. These effects are primarily mediated by the production of cyclic lipopeptides, particularly surfactin and iturin A, which act synergistically to disrupt fungal cell wall integrity. The broad-spectrum antimicrobial activity and multi-mechanistic mode of action of ZN-S18 underscore its potential as a promising biocontrol agent for managing tomato diseases. Future studies will focus on elucidating the molecular basis of the synergistic interaction between surfactin and iturin A and validating the efficacy of ZN-S18 under greenhouse and field conditions.

## 5. Conclusions

In this study, we isolated an endophytic *B. amyloliquefaciens* strain ZN-S18 from healthy tomato stems. ZN-S18 effectively suppressed Fol by inhibiting mycelial growth and spore germination, with the inhibitory effect on germination being irreversible. The antagonistic activity is primarily attributed to the production of cyclic lipopeptides, including surfactin and iturin A, which act synergistically to compromise Fol cell wall integrity. Moreover, ZN-S18 exhibits broad-spectrum activity against *B. cinerea* and *R. solanacearum*, while its VOCs further inhibit fungal growth and sporulation. In vivo assays confirmed that ZN-S18 cells, its culture filtrate, and the combination of surfactin and iturin A significantly reduced Fusarium wilt severity in tomato seedlings. Collectively, ZN-S18 represents a promising biocontrol agent for the sustainable management of tomato Fusarium wilt.

## Figures and Tables

**Table 1:** Primers used in this study.

Primer Name	Sequence (5′→3′)
For phylogenetic analysis	
27F	AGAGTTTGATCCTGGCTCAG
1492R	GGTTACCTTGTTACGACTT
rpoB_F	AGGTCAACTAGTTCAGTATGGAC
rpoB_R	AAGAACCGTAACCGGCAACTT
For verification of antagonistic agents	
Zwi_F	TTGGGGAAATATACAGCTCT
Zwi_R	GACCTTTTTGAAATGGGCGTA
BacD_F	GAAGGACACGGCGAGAGAGTC
BacD_R	CGCTGATGACTGTTTCATGCT
Itu_F	GATGCGGATCCTCTTGGATGT
Itu_R	ATCGTTCATGTGCTGCTTGAG
Bac_F	AAAAACAGTATTGGGYATCGCTGA
Bac_R	CCATGATGCCCTCKATRCTGAT
Sur_F	ACAGTATGGAGGCATGGTTC
Sur_R	TTCCGCCACTTTTTTCAGTT
Fen_F	TTTTGGCAGCAGGAAGAGTTT
Fen_R	GCTGTCGCTTCTGTTTTTTC

## Data Availability

All data generated or analyzed during this study are included in this published article and its Supplementary Information files.

## Supplementary Materials

The following supporting information can be downloaded at: https://www.mdpi.com/article/10.3390/jof12080576/s1, Figure S1: Phylogenetic analysis of ZN-S18; Figure S2: Selection of the optimal culture medium for antagonistic activity; Figure S3: Effects of ZN-S18 culture filtrate and lipopeptide treatments on Fusarium wilt development in tomato seedlings; Figure S4: Broad-spectrum antagonistic activity of ZN-S18 against other tomato pathogens.
