# Supplementary material for: An Endophytic Bacillus amyloliquefaciens ZN-S18 Suppresses Fusarium oxysporum f. sp. lycopersici Through Synergistic Activity of Surfactin and Iturin A
Source: J Fungi (Basel). 2026 Aug 4;12(8):576. doi: 10.3390/jof12080576 (PMC13514396; doi:10.3390/jof12080576)
Supplement: Supplementary file 1 [file jof-12-00576-s001.zip › jof-4443500-supplementary.pdf]

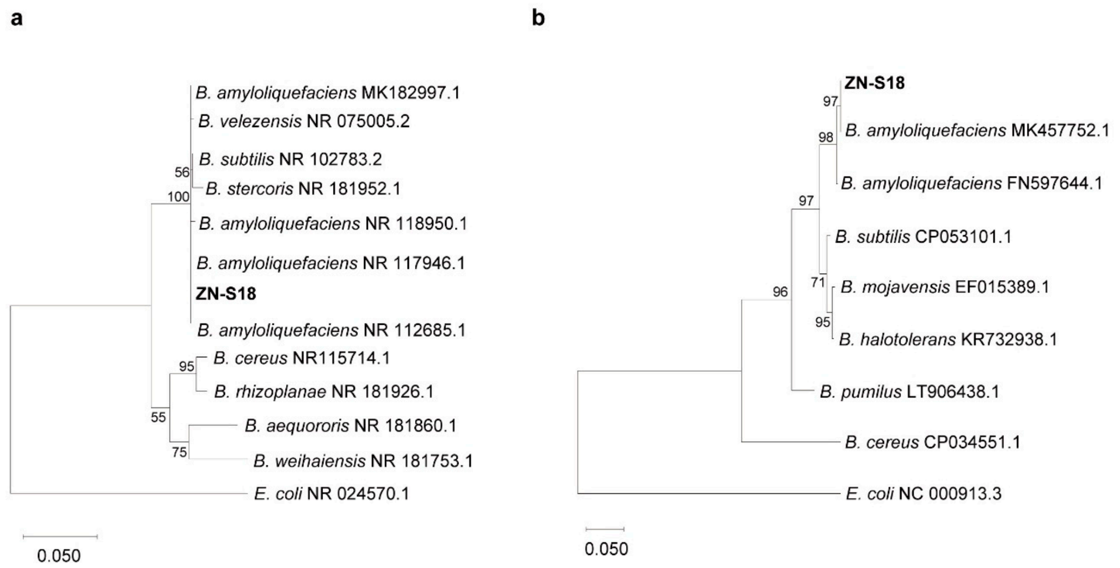

Figure S1 Phylogenetic analysis of ZN-S18. Phylogenetic trees were constructed based on (a) 16S rRNA and (b) *rpoB* gene sequences. Sequence alignment was performed using ClustalW in MEGA X, and the trees were generated using the maximum likelihood method with 1000 bootstrap replications.

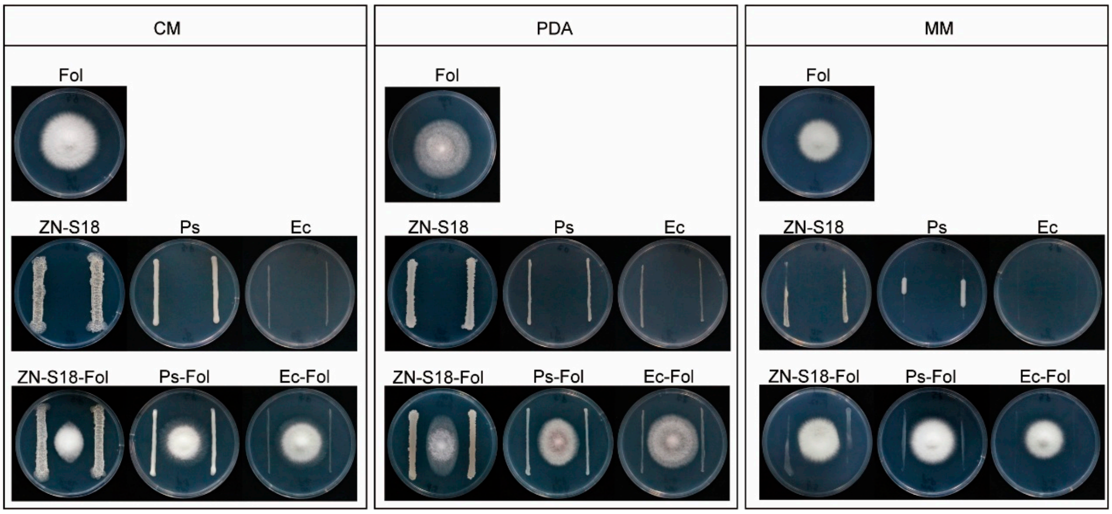

Figure S2 Selection of the optimal culture medium for antagonistic activity. Dual culture assays were performed on CM, PDA, and MM to identify the medium supporting the strongest antagonistic effect. Three-day-old *F. oxysporum* f. sp. *lycopersici* mycelial plugs were placed at the center of the plates, and endophytic bacteria were inoculated on both sides. Plates were incubated at 25°C for 3 d. *Pseudomonas syringae* and *Escherichia coli* were used as controls. Fol, *F. oxysporum* f. sp. *lycopersici*; Ps, *P. syringae*; Ec, *E. coli*.

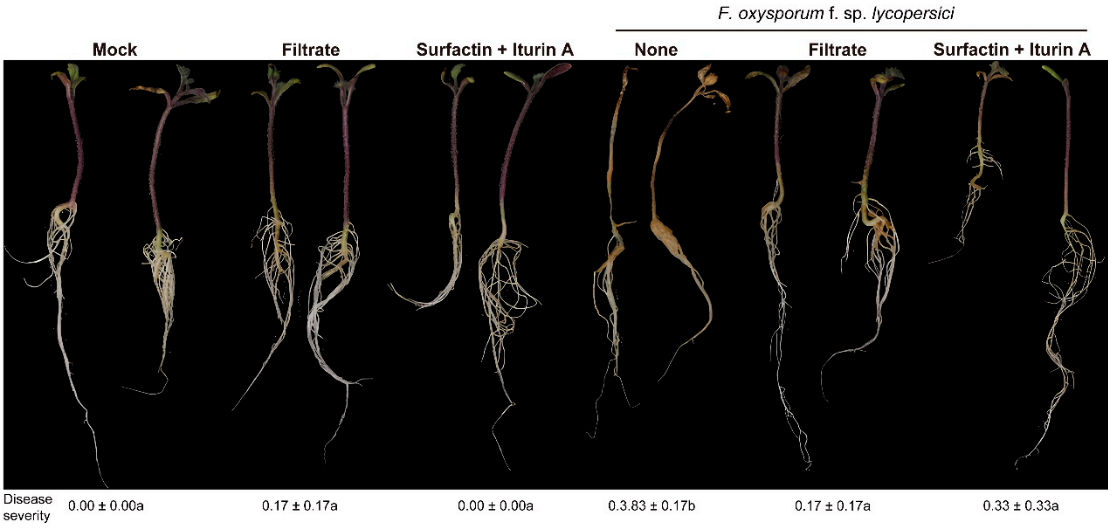

Figure S3 Effects of ZN-S18 culture filtrate and lipopeptide treatments on Fusarium wilt development in tomato seedlings. Tomato seedlings were prepared as described in Fig. 2. Prior to Fol inoculation, seedlings were pretreated with ZN-S18 culture filtrate, a combination of surfactin and iturin A, or sterile water. Two days later, seedlings were inoculated with Fol spore suspension (1 × 10^6 spores/mL). Disease symptoms were recorded after 30 d. The experiment was repeated three times with two replicates per treatment. Error bars represent the mean ± standard error (n = 3). Different letters indicate significant differences based on one-way ANOVA followed by Tukey's HSD test (P < 0.05).

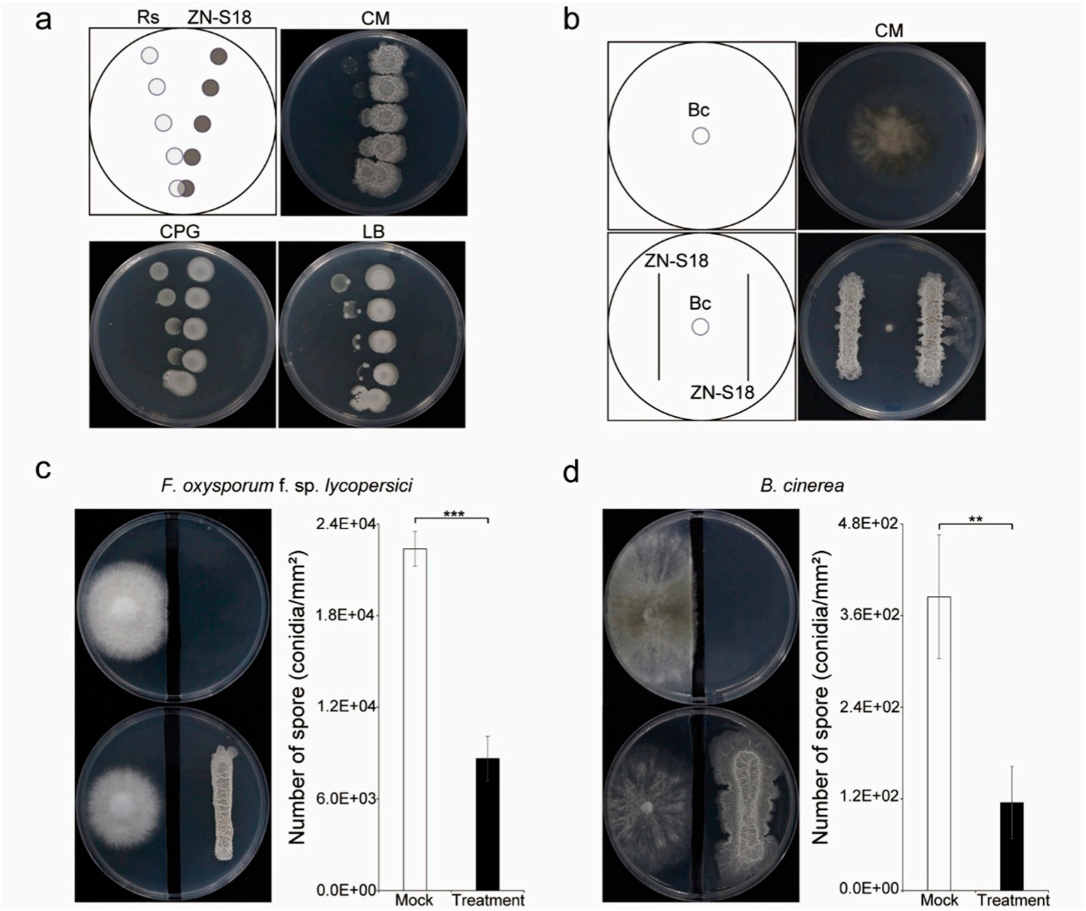

Figure S4 Broad-spectrum antagonistic activity of ZN-S18 against other tomato pathogens. (a, b) Dual culture assays showing antagonistic activity against *Ralstonia solanacearum* on CM, CPG, and LBA, and against *Botrytis cinerea* on CM. Co-cultures were incubated at 28°C (*R. solanacearum*) or 25°C (*B. cinerea*) for 2 or 3 d, respectively. (c, d) Effects of volatile organic compounds produced by ZN-S18 on mycelial growth and spore production of *F. oxysporum* f. sp. *lycopersici* and *B. cinerea*. Partitioned plates with a 1 cm gap were used to prevent direct contact between the endophyte and the fungal pathogens. After sealed incubation at 25°C for 4 d, fungal plugs were collected, vortexed in sterile water, and spore concentrations were quantified using a hemocytometer. The experiment was repeated three times with five replicates per treatment. Error bars represent the standard error. Asterisks indicate significant differences relative to the mock treatment (**$P < 0.01$, ***$P < 0.001$).
